# Survival from cancer of the ovary in England and Wales up to 2001

**DOI:** 10.1038/sj.bjc.6604593

**Published:** 2008-09-23

**Authors:** N Cooper, M J Quinn, B Rachet, E Mitry, M P Coleman

**Affiliations:** 1 Social and Health Analysis and Reporting Division, Office for National Statistics (Room FG/114), 1 Myddelton Street, London EC1R 1UW, UK; 2 Cancer Research UK Cancer Survival Group, Non-Communicable Disease Epidemiology Unit, Department of Epidemiology and Population Health, London School of Hygiene and Tropical Medicine, Keppel Street, London WC1E 7HT, UK; 3 Département d'Hépatogastroentérologie et Oncologie Digestive, Centre Hospitalo-Universitaire Ambroise-Paré, 9 avenue Charles de Gaulle, F-92100 Boulogne, France

The incidence of ovarian cancer varies widely around the world, with the highest rates in the developed countries of Europe and North America, and the lowest in Africa and Asia (Parkin *et al*, 2002). In England and Wales, ovarian cancer is the most common gynaecological cancer, and the fourth most common after cancers of the breast, large bowel and lung, representing some 5% of all cancers in women. It is rare in premenopausal women, with less than 10% of cases arising under the age of 45.

The pattern of incidence for ovarian cancer with age is similar to that of breast and uterine cancers, and they share some reproductive risk factors. Late menarche, high parity, early menopause and long-term use of combined oral contraceptives all confer a lower risk of ovarian cancer, probably by reducing the number of ovulatory cycles.

Incidence in England and Wales has increased gradually since the early 1970s, especially in older women (Coleman *et al*, 1993), but the upward trend in younger women appears to have reached a plateau by the late 1990s (Quinn *et al*, 2001). Incidence is 5–10% higher among women in more affluent groups than in the most deprived group, and it increased further in these groups during the 1990s (data not shown). In the late 1990s, about 6000 women were diagnosed with ovarian cancer in England and Wales each year.

Ovarian cancer accounts for 4000 deaths a year in England and Wales, about 6% of all cancer deaths in women. In contrast to the increasing trend in incidence, overall age-standardised mortality has fallen slightly over the last decade, but a 20–30% fall in mortality among women under 65 years has been balanced by an increase of about 10% in mortality among older women. Survival from ovarian cancer is the lowest among the gynaecological cancers, because it is often at an advanced stage when diagnosed.

Some 81 600 women were registered with an ovarian tumour in England and Wales during the 14-year period 1986–1999, but more than 6000 of these tumours were benign, of uncertain behaviour, or metastatic to the ovary from a primary malignancy elsewhere. Of the 75 800 women resident in England and Wales who were registered with a primary, malignant tumour of the ovary, some 63 800 were included in the analyses (84% of those eligible). One percent of women were excluded because their vital status was not known on 5 November 2002, when the data were extracted for analysis and 10% because their recorded survival time was zero (mainly death certificate only (DCO) cases whose survival time was unknown). A further 3% were excluded because it was not the woman's first primary, invasive cancer, a previous malignancy having been registered for the same woman at some time since 1971. The proportion of cases excluded from analysis as DCOs did not vary by year of diagnosis or by deprivation category.

Tumours of the ovary have usually been grouped with those of the Fallopian tube, broad ligament and other uterine adnexa (ICD-9 183, ICD-10 C56–57). Ovarian tumours were only assigned a separate three-digit rubric in the International Classification of Diseases with the introduction of the tenth revision in 1995 (ICD-9 183.0; ICD-10 C56), but in any case almost 99% of the tumours were coded to the ovary, with only about 1% to the Fallopian tube (183.2, C57.0); tumours coded to the broad ligament and other adnexa were extremely rare. As is conventional, therefore, these tumours were included with ovarian cancers in the survival analyses, for consistency in the interpretation of long-term trends.

Adenocarcinomas accounted for most tumours that were assigned to a specific morphology, with 39% coded to serous, papillary or mucinous cystadenocarcinoma and 34% to other specific types of adenocarcinoma; 20% were poorly specified carcinomas.

## Survival trends

Relative survival at 1 year rose from 56% for women diagnosed during 1986–1990 to 66% for those diagnosed during 1996–1999. This represents a rapid and significant deprivation-adjusted increase of about 5% every 5 years (Table 1). Five-year survival rose from 30 to 38%, representing a statistically significant increase of 3% every 5 years.

**Figure 1:**
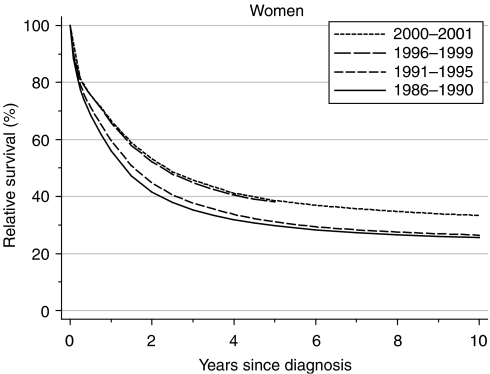
Relative survival (%) up to 10 years after diagnosis by calendar period of diagnosis: England and Wales, adults (15--99 years) diagnosed during 1986--1999 and followed up to 2001. Survival estimated with cohort or complete approach (1986--1990, 1991--1995, 1996--1999) or hybrid approach (2000--2001) (see Rachet *et al*, 2008).

Short-term predictions based on hybrid analysis (Brenner and Rachet, 2004) of patients' survival experience during 2000–2001 suggest that the underlying increase in survival up to 5 years is likely to continue (Table 1, Figure 1).

It is striking that relative survival at 10 years has remained low, around 26%, for women who were diagnosed in both the late 1980s and the early 1990s (Table 1, Figure 1). These estimates are based on classical techniques of following up all or most of the women diagnosed in those periods for up to 10 years, and no such estimate can yet be made for women diagnosed during 1996–1999. In this case, however, hybrid analysis based on events during 2000–2001 suggests that a substantial increase in 10-year survival can be expected within the next 5 years or so, to approximately 33% (Figure 1). This is because 5-year survival increased much more quickly between the early and late 1990s than over the previous 5 years (Table 1, Figure 1), and the hybrid estimate of 10-year survival incorporates this recent jump in medium-term survival.

## Deprivation

**Figure 2:**
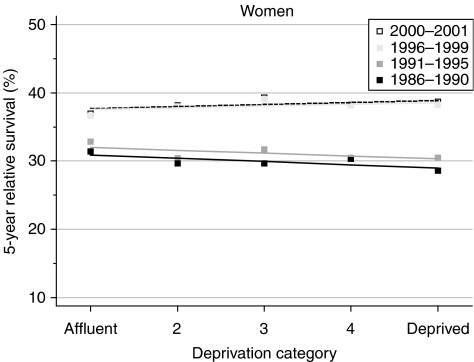
Trends in the deprivation gap in 5-year relative survival (%) by calendar period of diagnosis: England and Wales, adults (15--99 years) diagnosed during 1986--1999 and followed up to 2001.

Women living in more deprived areas who were diagnosed with ovarian cancer in the late 1980s had lower survival than more affluent women at both 1 and 5 years after diagnosis (Table 2). The deprivation gap in survival between the affluent and deprived was significantly wider at 1 year (−5.1%) than at 5 years (−1.9%). Survival rose slightly more for women in the deprived categories than for the more affluent groups. The deprivation gap in 1-year survival fell slightly to −4.6%, and the deprivation gap in 5-year survival disappeared, becoming positive (1.0%). These changes are small, and not individually significant, but they appear systematic. It is notable that the larger increase in 5-year survival in the late 1990s (Figure 1) is accompanied by a distinct flattening of the deprivation gradient (Figure 2).

Short-term predictions of the socioeconomic gradient in ovarian cancer survival suggest little imminent change (Table 2), with a significant deficit in 1-year survival of −5% for the least affluent women, but little difference in longer-term survival (Table 2).

## Comment

The more recent improvement in survival is probably due to progress in treatment. Only a minority of women are diagnosed early enough for radical hysterectomy and oophorectomy alone to be successful, and many women are given adjuvant chemotherapy. Regimens combining taxanes (paclitaxel and docetaxel) with platinum compounds are now generally recommended as first-line treatment for women with advanced disease (Lister-Sharp *et al*, 2000). Evidence of effectiveness suggests that survival is improved when surgery is done by specialists in gynaecological oncology and when treatment is undertaken by expert multi-disciplinary teams (NHS Centre for reviews and dissemination, 1999).

Survival at 1 year after diagnosis is lower for women living in deprived areas: these women tend to have more advanced disease at diagnosis (Brewster *et al*, 2001). However, the gradual disappearance of the deprivation gap in 5-year survival by the late 1990s – in contrast to the pattern for most other cancers in women – suggests that deprived women diagnosed during 1996–1999 generally obtained at least as good access to optimal treatment as affluent women.

Thus among the 17 different cancers in women examined in these analyses, ovarian cancer was one of only five (with stomach, pancreas, kidney, and the different types of leukaemia) for which survival for women living in the poorest one-fifth of electoral wards in England and Wales in the late 1990s was not lower than for women in the richest fifth of wards. The proportion of DCO cases was similar in each year and in each deprivation category (data not shown); therefore, estimates of trend in the deprivation gradient in survival are not likely to be biased as a result of their exclusion from analysis. The deprivation-specific life tables used in the analyses are the same as those used for all other cancers, for most of which the deprivation gap in survival widened during the 1990s. Narrowing of the deprivation gap in survival from ovarian cancer thus seems unlikely to be an artefact either of the data or of our analytic approach.

Advanced stage at diagnosis frequently limits the potential for radical treatment. Evidence from a large randomised controlled trial indicates that earlier detection by screening does reduce mortality (Jacobs *et al*, 1999). Mass screening for ovarian cancer is being evaluated in the United Kingdom Collaborative Trial of Ovarian Cancer Screening (UKCTOCS) (Lewis and Menon, 2003). This trial will compare two types of screening test (serum CA125 and transvaginal ultrasound) with no test at all; it is due to end in 2010.

Compared with many other common cancers, survival from ovarian cancer did not improve greatly during the 1980s (Coleman *et al*, 1999). For women diagnosed in England and Wales during the early 1990s, 5-year survival from ovarian cancer was still 5–8% below the age-standardised European average relative survival of 37% (Sant *et al*, 2003). It remains to be seen whether the improvement in survival during the late 1990s will have narrowed the gap with the European average survival.

## Figures and Tables

**Table 1:** Trends in relative survival (%) by time since diagnosis and calendar period of diagnosis: England and Wales, adults (15–99 years) diagnosed during 1986–1999 and followed up to 2001

		**Calendar period of diagnosis^a^**						**Average change (%)**		**Prediction^c^ for patients**	
		**1986–1990**		**1991–1995**		**1996–1999**		**every 5 years^b^**		**diagnosed during 2000–2001**	
**Time since diagnosis**		**Survival (%)**	**95% CI**	**Survival (%)**	**95% CI**	**Survival (%)**	**95% CI**	**Survival (%)**	**95% CI**	**Survival (%)**	**95% CI**
1 year	Women	**56.0**	(55.2, 56.6)	**59.6**	(59.0, 60.3)	**65.8**	(65.1, 66.5)	4.9**	(3.6, 6.3)	**66.6**	(65.6, 67.5)
5 years	Women	**29.8**	(29.1, 30.4)	**31.1**	(30.4, 31.7)	**38.1**	(37.3, 38.9)	2.9**	(1.5, 4.3)	**38.6**	(37.6, 39.6)
10 years	Women	**25.6**	(24.9, 26.2)	**26.4**	(25.7, 27.1)			**−0.3**	(−2.7, 2.2)	**33.3**	(32.2, 34.4)

CI=confidence interval.

a Survival estimated with cohort or complete approach (see Rachet *et al*, 2008).

b Mean absolute change (%) in survival every 5 years, adjusted for deprivation (see Rachet *et al*, 2008).

c Survival estimated with hybrid approach (see Rachet *et al*, 2008).

^**^P<0.01.

**Table 2:** Trends in the deprivation gap in relative survival (%) by time since diagnosis and calendar period of diagnosis: England and Wales, adults (15–99 years) diagnosed during 1986–1999 and followed up to 2001

		**Calendar period of diagnosis^a^**						**Average change (%)**		**Prediction^c^ for patients**	
		**1986–1990**		**1991–1995**		**1996–1999**		**every 5 years^b^**		**diagnosed during 2000–2001**	
**Time since diagnosis**		**Deprivation gap (%)**	**95% CI**	**Deprivation gap (%)**	**95% CI**	**Deprivation gap (%)**	**95% CI**	**Deprivation gap (%)**	**95% CI**	**Deprivation gap (%)**	**95% CI**
1 year	Women	−5.1**	(−7.2, −3.1)	−4.2**	(−6.1, −2.3)	−4.6**	(−6.6, −2.7)	**0.3**	(−1.2, 1.7)	−5.1**	(−7.8, −2.3)
5 years	Women	**−1.9**	(−3.8, 0.1)	**−1.7**	(−3.5, 0.2)	**1.0**	(−1.4, 3.5)	**1.4**	(−0.2, 3.0)	**1.2**	(−1.8, 4.2)
10 years	Women	**−1.1**	(−3.0, 0.8)	**0.2**	(−1.8, 2.2)			**1.3**	(−1.5, 4.1)	**2.7**	(−0.4, 5.9)

CI=confidence interval.

a Survival estimated with cohort or complete approach (see Rachet *et al*, 2008).

b Mean absolute change (%) in the deprivation gap in survival every 5 years, adjusted for the underlying trend in survival (see Rachet *et al*, 2008).

c Survival estimated with hybrid approach (see Rachet *et al*, 2008). ^**^P<0.01.
